# Supplementary material for: Neural dynamics of proactive and reactive cognitive control in medial and lateral prefrontal cortex
Source: bioRxiv. 2025 Feb 13:2025.02.12.637987. Preprint. [Version 1] doi: 10.1101/2025.02.12.637987 (PMC11844492; doi:10.1101/2025.02.12.637987)
Supplement: 1 [file NIHPP2025.02.12.637987V1-supplement-1.pdf]

bioRxiv preprint doi: https://doi.org/10.1101/2025.02.12.637987; this version posted February 13, 2025. The copyright holder for this preprint (which was not certified by peer review) is the author/funder, who has granted bioRxiv a license to display the preprint in perpetuity. It is made available under aCC-BY 4.0 International license.

Supplemental Introduction

Cognitive control adjustments are commonly studied through between-trial adaptations. Foundational fMRI work demonstrates dmPFC activity on conflict trials predicts behavioral adjustments on the next trial[1,2], the next trial's adjustments correspond with greater dlPFC activation[1,2], and this dlPFC activation is correlated with the previous trial's dmPFC activation[1]. Recent human iEEG studies found that neurons in the anterior cingulate fire faster after conflict than after non-conflict trials[3], and post-conflict dlPFC sustained HFA has been shown to correlate with longer reaction times (RTs)[4]. Although between-trial adaptations cannot disentangle the contributions of proactive and reactive control and are thus not the core focus of this study, we sought to connect our main findings to prior results by reporting supplemental behavioral and neural results on post-conflict adaptations.

Supplemental Results

Between-trial behavioral adaptations after conflict

We found a main effect of previous trial conflict, where RTs were slower after Conflict trials compared to after NoConflict trials (t_{5860}=5.63, p=1.90 x 10^{-08}). The interaction between current and previous conflict was also significant (t_{5860}=-2.19, p=0.03), a phenomenon described previously as the Gratton effect [5]. Post-hoc, pairwise comparisons revealed that the Stroop effect was largest when the previous trial was NoConflict (t_{5860}=34.3, p=0, Tukey corrected), with a smaller but still significant effect when the previous trial was Conflict (t_{5860}=20.3, p=2.15 x 10^{-12}). The Gratton effect was driven by slower RTs on current NoConflict trials when the previous

bioRxiv preprint doi: https://doi.org/10.1101/2025.02.12.637987; this version posted February 13, 2025. The copyright holder for this preprint (which was not certified by peer review) is the author/funder, who has granted bioRxiv a license to display the preprint in perpetuity. It is made available under aCC-BY 4.0 International license.

trial was Conflict (t_{5860}=5.63, p=1.14 x 10^{-07}, Figure S1), whereas Conflict trials were not affected by previous trial conflict (t_{5860}=1.32, p=0.549).

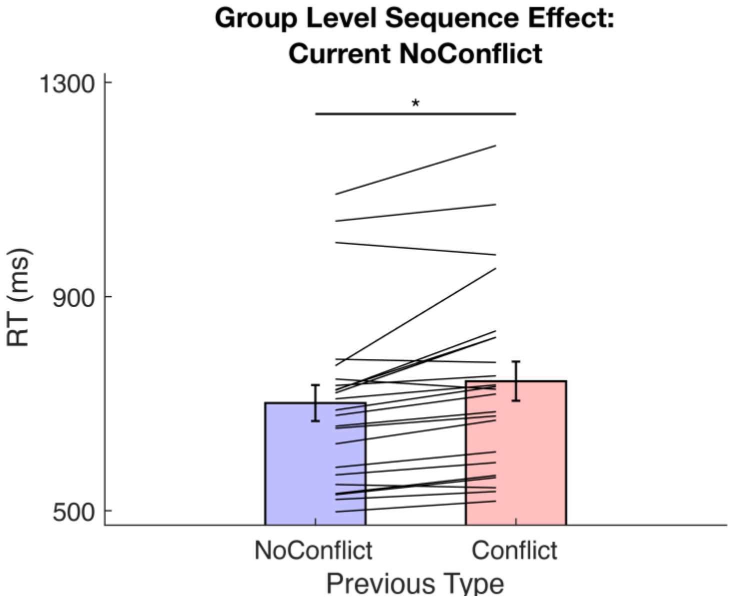

Figure S1. Conflict prolongs response times on subsequent NoConflict Trials. Bar graphs depicting mean RT across subjects with individual subject mean RTs shown as lines. RTs were taken only from current NoConflict trials and plotted as a function of previous conflict.

dmPFC theta tracks post-conflict slowing

We examined the neural basis of these within-trial reactive control dynamics by examining the neural basis of post-conflict slowing on NoConflict trials. While we did not find condition-specific differences in neural activity in either frequency band or either region based on previous Conflict versus NoConflict trials, we found a relationship between theta power and RT adjustments that was specific to dmPFC and the condition with behavioral slowing after conflict. Specifically, higher RTs (greater adjustments) predicted lower dmPFC theta power in the early stage of NoConflict trials following Conflict trials (LMM, all p<=0.021, Figure S2A).

bioRxiv preprint doi: https://doi.org/10.1101/2025.02.12.637987; this version posted February 13, 2025. The copyright holder for this preprint (which was not certified by peer review) is the author/funder, who has granted bioRxiv a license to display the preprint in perpetuity. It is made available under aCC-BY 4.0 International license.

Importantly, this effect of RTs on theta was absent on post-NoConflict trials without control adaptations (LMM, all p>=0.05, Figure S2C), and no RT adjustment effects were observed in dlPFC theta (Figure S2B, D). During the later, peri-response window, dmPFC theta power showed a general relationship with RTs on both post-Conflict and post-NoConflict trials, where trials with longer RTs exhibited greater theta power (LMM, all p<=0.01, Figure S2A, C), which is consistent with proposals linking dmPFC theta to reactive monitoring of control demands[6].

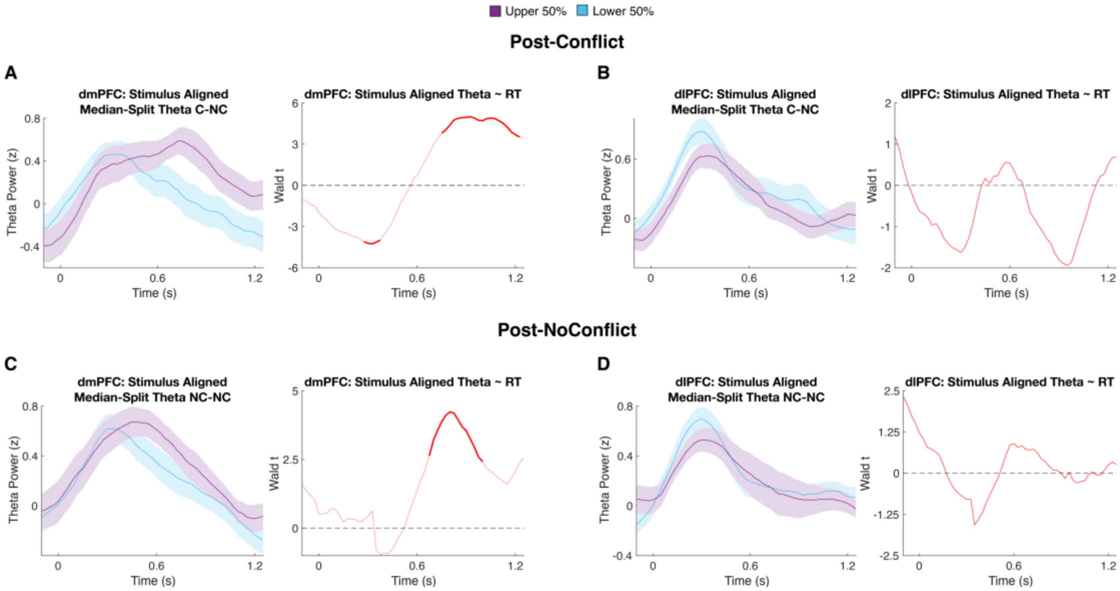

Figure S2. dmPFC theta power indexes between-trial control adjustments. *A*, (Left) Stimulus-locked dmPFC theta power averaged for slow (purple) and fast (blue) RTs based on a median split of RTs on NoConflict trials preceded by Conflict. Statistics are based on LMM regressions, median split for visualization purposes only. (Right) Wald t time course from the LMM with RT predicting theta power on Conflict->NoConflict trials. *B*, Same as in *A* but for dlPFC. *C*, Same as in *A* but for NoConflict->NoConflict trials. *D*, Same as in *C* but for dlPFC.

Response-locked oscillatory and population dynamics vary with conflict anticipation

To determine whether the theta, beta, and HFA conflict signals were best interpreted as stimulus- or response-driven, we repeated our main analysis investigating the interplay between

bioRxiv preprint doi: https://doi.org/10.1101/2025.02.12.637987; this version posted February 13, 2025. The copyright holder for this preprint (which was not certified by peer review) is the author/funder, who has granted bioRxiv a license to display the preprint in perpetuity. It is made available under aCC-BY 4.0 International license.

proactive and reactive control by realigning our data to the response. We largely found consistent effects with stimulus-aligned data except for dmPFC theta and HFA, both of which were trending and visually apparent but did not reach statistical significance (Figure S3).

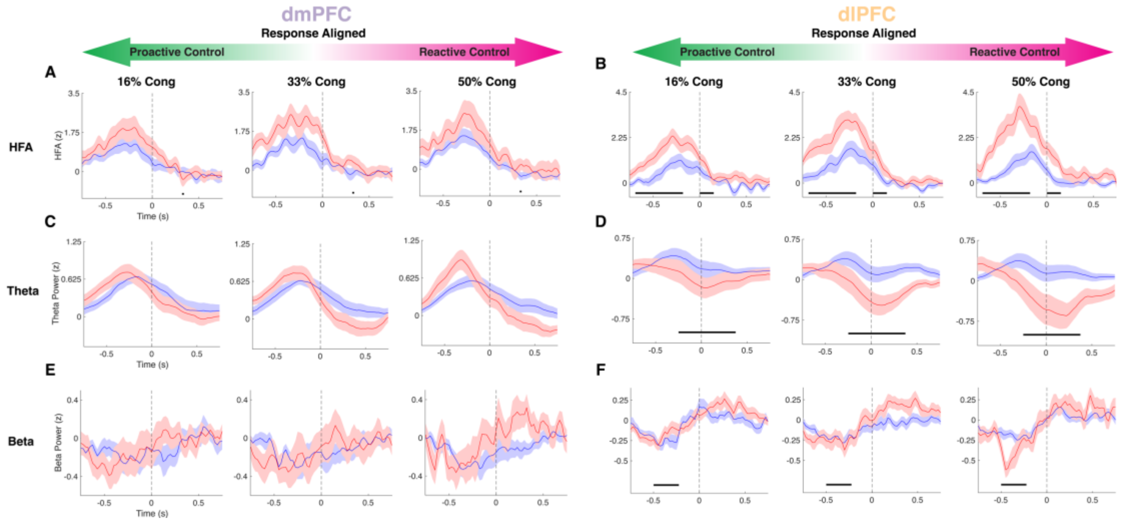

Figure S3. Bi-directional modulation of response-locked neural conflict signals by proactive control. *A*, dmPFC HFA in Conflict (red) and NoConflict (blue) trials across 3 block types, from highest to lowest proactive control (left to right: 16%, 33%, 50% congruent). *B*, Same as in *A* but for dlPFC. *C*, *D* Same as *A*, *B* but for theta power. *E*, *F* Same as above but for beta power. Horizontal black lines indicate significance for the CurrentConflict:%Cong interaction. Vertical dashed lines indicate the RT.

Supplemental Discussion

On the trial-by-trial timescale, we observed classical post-conflict slowing on NoConflict trials[5], which is typically interpreted as increased caution after difficult responses. Ultimately, we did not find condition-specific differences in neural power related to previous trial type. Instead, we found that early trial dmPFC theta power was reduced on NoConflict trials with stronger post-conflict slowing, which was specific to this region and condition. This reduction in the early dmPFC theta response may reflect a reduced need to recruit control resources due to prior recruitment on previous Conflict trials. Regardless, the selectivity of this relationship to conditions showing trial-by-trial behavioral adjustments aligns with our finding that pre-trial

bioRxiv preprint doi: https://doi.org/10.1101/2025.02.12.637987; this version posted February 13, 2025. The copyright holder for this preprint (which was not certified by peer review) is the author/funder, who has granted bioRxiv a license to display the preprint in perpetuity. It is made available under aCC-BY 4.0 International license.

dmPFC theta increases with proactive control to support the theory that dmPFC theta power plays a key role in managing control over multiple time scales[7].

References

1. Kerns, J. G. *et al.* Anterior Cingulate Conflict Monitoring and Adjustments in Control. *Science* **303**, 1023–1026 (2004).

2. Kerns, J. G. Anterior cingulate and prefrontal cortex activity in an FMRI study of trial-to-trial adjustments on the Simon task. *NeuroImage* **33**, 399–405 (2006).

3. Sheth, S. A. *et al.* Human Dorsal Anterior Cingulate Cortex Neurons Mediate Ongoing Behavioral Adaptation. *Nature* **488**, 218–221 (2012).

4. Bartoli, E. *et al.* Temporal Dynamics of Human Frontal and Cingulate Neural Activity During Conflict and Cognitive Control. *Cereb Cortex* **28**, 3842–3856 (2018).

5. Gratton, G., Coles, M. G. & Donchin, E. Optimizing the use of information: strategic control of activation of responses. *J Exp Psychol Gen* **121**, 480–506 (1992).

6. Shenhav, A., Botvinick, M. M. & Cohen, J. D. The expected value of control: An integrative theory of anterior cingulate cortex function. *Neuron* **79**, 217–240 (2013).

7. Chinn, L. K., Pauker, C. S. & Golob, E. J. Cognitive control and midline theta adjust across multiple timescales. *Neuropsychologia* **111**, 216–228 (2018).

8. Lachaux, J.-P. *et al.* Estimating the time-course of coherence between single-trial brain signals: an introduction to wavelet coherence. *Neurophysiol Clin* **32**, 157–174 (2002).
